# Pilot Point-of-Care Ultrasound Curriculum at Harvard Medical School: Early Experience

**DOI:** 10.5811/westjem.2016.8.31387

**Published:** 2016-09-12

**Authors:** Joshua S. Rempell, Fidencio Saldana, Donald DiSalvo, Navin Kumar, Michael B. Stone, Wilma Chan, Jennifer Luz, Vicki E. Noble, Andrew Liteplo, Heidi Kimberly, Minna J. Kohler

**Affiliations:** * Harvard Medical School, Boston, Massachusetts; † Brigham and Women’s Hospital, Department of Emergency Medicine, Boston, Massachusetts; ‡ Brigham and Women’s Hospital, Department of Cardiovascular Medicine, Boston, Massachusetts; § Brigham and Women’s Hospital, Department of Internal Medicine, Boston, Massachusetts; ¶ Brigham and Women’s Hospital, Department of Radiology, Boston, Massachusetts; || Harvard Medical School, Department of Physical Medicine and Rehabilitation, Spaulding Rehabilitation Hospital, Charlestown, Massachusetts; # Massachusetts General Hospital, Department of Emergency Medicine, Boston, Massachusetts; ** Harvard Medical School, Department of Medicine, Division of Rheumatology, Allergy, Immunology, Boston, Massachusetts

## Abstract

**Introduction:**

Point-of-care ultrasound (POCUS) is expanding across all medical specialties. As the benefits of US technology are becoming apparent, efforts to integrate US into pre-clinical medical education are growing. Our objective was to describe our process of integrating POCUS as an educational tool into the medical school curriculum and how such efforts are perceived by students.

**Methods:**

This was a pilot study to introduce ultrasonography into the Harvard Medical School curriculum to first- and second-year medical students. Didactic and hands-on sessions were introduced to first-year students during gross anatomy and to second-year students in the physical exam course. Student-perceived attitudes, understanding, and knowledge of US, and its applications to learning the physical exam, were measured by a post-assessment survey.

**Results:**

All first-year anatomy students (n=176) participated in small group hands-on US sessions. In the second-year physical diagnosis course, 38 students participated in four sessions. All students (91%) agreed or strongly agreed that additional US teaching should be incorporated throughout the four-year medical school curriculum.

**Conclusion:**

POCUS can effectively be integrated into the existing medical school curriculum by using didactic and small group hands-on sessions. Medical students perceived US training as valuable in understanding human anatomy and in learning physical exam skills. This innovative program demonstrates US as an additional learning modality. Future goals include expanding on this work to incorporate US education into all four years of medical school.

## INTRODUCTION

The use of point-of-care ultrasound (POCUS), or bedside ultrasound, has expanded across many medical and surgical specialties.^1^ While ultrasound has a traditional role in radiology, obstetrics-gynecology, and cardiology, advances in technology have facilitated the integration of POCUS into a wider variety of fields such as emergency medicine, critical care, anesthesia, and rheumatology, among others.^2^ Incorporation of POCUS training into post-graduate medical education has increased and it is now a component of emergency medicine residency that is required by the Accreditation Council for Graduate Medical Education.^3^

As focused ultrasonography takes a more prominent role in medical care, there is increasing interest in introducing it earlier at the undergraduate medical education level. Multiple reports to date describe the feasibility of introducing US into medical school curricula. Such efforts have been well received by students who report a high level of satisfaction with ultrasonography as well as interest in additional training and incorporation of bedside US during medical school education. Efforts have also shown that POCUS introduced during anatomy and the physical exam course show promise to increase students’ knowledge.^4^–^16^

A 2014 report by Bahner et al described the state of ultrasound education in U.S. medical schools. In 82/143 medical schools that responded to the survey, 62.2% reported some level of US training in their medical education curriculum. The majority of respondents (78.9%) agreed that US should be part of the undergraduate medical education though only 18.6% reported it was a priority at their institution.^17^ A few schools have reported on their successful experiences of integrating US into a vertical four-year medical school curriculum.^18^–^20^ To date, fully developed POCUS programs are limited to a small number of medical schools and there are no national guidelines as use of bedside ultrasound spreads into additional medical student curriculum.

### Objectives

The objectives of our study were the following: 1) determine the feasibility and barriers of integrating a POCUS curriculum into the first- and second-year medical school curriculum at our institution and 2) determine student-perceived values and attitudes toward point-of-care ultrasonography in the medical school curriculum.

## METHODS

This was a pilot study to assess the feasibility and student response of introducing bedside ultrasonography into the existing curriculum during the 2013–14 academic year. A multi-disciplinary team of instructors represented by emergency medicine, radiology, internal medicine, anatomy and physiology, cardiology, pediatrics, rheumatology, and physical medicine and rehabilitation contributed to the development and integration of a new POCUS curriculum.

### Curriculum development

Before initiation of this pilot ultrasound curriculum, student exposure to US was limited. Many students were unaware that US was being used as an educational tool at other medical schools. A core group of multi-disciplinary faculty with ultrasound expertise (JR, DD, MJK) met with the anatomy and physical diagnosis course directors (TV, CM, FS) to create a set of potentially feasible educational objectives based on the allotted time that was provided for the pilot US sessions. This group created an outline and reading materials to provide students prior to each scheduled session, structured the didactic and hands-on components of the sessions, identified and organized multi-disciplinary POCUS instructors and clinical instructors across all four affiliated teaching hospitals to be available for these sessions, and arranged US equipment access. This group created post-curriculum surveys to obtain student feedback after the sessions. “Train-the-trainer” sessions to standardize teaching by residents and fellows to faculty level teaching were also provided (JR, MJK) prior to each medical student session. The hands-on sessions were primarily taught by resident and fellow physicians with significant oversight by a core group of attending-level physicians. Faculty representation from each discipline varied depending on the topic; for instance, abdominal sessions were largely taught by faculty in emergency medicine and radiology, while musculoskeletal sessions were taught primarily by faculty from internal medicine, emergency medicine, and rheumatology.

### Ultrasound into the first-year anatomy course

We introduced US into the first-year anatomy class during the 2013–14 year. A 40-minute introductory lecture to the class using case-based examples and a basic introduction to US was followed by four hands-on ultrasound sessions. Sessions included basic anatomy of the neck, vascular structures, thorax, cardiac system, abdomen, and musculoskeletal. These sessions were held over a three-month period during the anatomy course. The hands-on sessions were held at the same time and in parallel with the gross dissection lab. Groups of 4–6 students rotated through 10–15 minute hands-on US sessions. The sessions were run in a separate space of the anatomy lab. One student in each group acted as a model while the remaining students acquired focused images of the anatomic structures being dissected during the lab session. US instructors ranged from resident to attending-level physicians from a variety of specialties. Learning objectives were distributed to instructors prior to each session. US teaching sessions for the resident level instructors, prior to student teaching, were conducted to ensure a high level of quality and consistency among instructors. Given the limited allotted time for each station, a checklist of certain anatomical structures and their ultrasound orientation views were emphasized in the short stations. Table 1 provides a brief overview of the anatomy labs sessions.

### Second-year physical diagnosis course

At our institution, second-year students are divided among the four affiliated teaching hospitals for a year-long course in the physical exam. This pilot curriculum took place at one of the four designated course sites and included all 38 students at that single site. During the first half of the course, four four-hour sessions were held: 1) introduction to ultrasound; 2) the evaluation of the neck and thyroid; 3) the musculoskeletal exam; and 4) the abdominal exam. Each session started with a brief didactic session (10–15 minutes) with the majority of the time spent on hands-on instruction. Students were divided into groups of four and physical exam skills were taught in parallel with ultrasonographic correlation. Instructors taught physical exam skills along with US skills including image acquisition, interpretation, and correlation into the physical exam. Clinical instructors who were able to teach physical exam skills but unable to teach the ultrasound skills portion were paired with an ultrasound instructor who provided the US teaching. Learning objectives were distributed to instructors prior to each session. Ultrasound and physical exam teaching sessions for the resident-level instructors, prior to student teaching, were held to standardize a high level of quality among instructors. Table 2 shows the focused goals of each session and the content that was covered.

Students completed a post-curriculum survey of the US sessions to determine the perceived value and attitudes toward the sessions. Survey assessment was obtained using a five-point Likert scale (1, strongly disagree; 5, strongly agree), and results are reported as means with standard deviation.

### Second-year ultrasound selective

Additionally, an advanced session was offered to students during the second half of the physical exam course. Students are offered a variety of “selectives” during the spring of the physical exam course meant to prepare them for their clinical rotations. An ultrasound “selective” was offered to students during the 2013–14 year. This was offered to the same subset of students who took part in the US sessions as part of the physical diagnosis course. A total of 12 students participated in the advanced US session. This session was offered four times during the course to keep the student-to-instructor ratio low. Each three-hour session started with a brief lecture reviewing basics of US machine image acquisition and orientation. Students were subsequently introduced to the focused assessment with sonography in trauma (FAST) examination. Following the didactic portion, the instructor took students to the emergency department where the small groups incorporated basic abdominal and cardiac imaging into the history and physical exam of a patient volunteer. Students completed a brief pre- and post-curriculum survey meant to assess knowledge acquired as well as overall experience and satisfaction with the advanced session. Students were assessed on such questions as listing the basic views of the FAST exam, identifying basic cardiac views, cardiac chambers, as well as very basic questions on US physics and the appearance of fluid on US.

### Ethics

This study was deemed to be non-human research by the Harvard Medical School Institutional Review Board and was approved by the Harvard Medical School Academy.

## RESULTS

### First-year anatomy course

All first-year anatomy students (n=176) participated in the lab sessions. The short hands-on sessions proved to be a feasible addition to the course and 91% of students agreed or strongly agreed that the ultrasound sessions were a positive addition to the course.

### Second-year physical exam course

Thirty-three out of a total of 38 students (87% response rate) completed a post-assessment survey of the US sessions. The post-assessment survey was distributed immediately after the session and it is unclear why five surveys were not completed or went missing. Using a five-point Likert scale, 94% of students either agreed or strongly agreed with the statement that they would like to see US incorporated into the medical school curriculum. Eighty-five percent of students agreed or strongly agreed that they would benefit from expanded ultrasound experience during all four years of medical school, and 97% of students agreed or strongly agreed that it is important for them to learn basic US skills during medical school. Eighty-eight percent of students agreed or strongly agreed that the US sessions both allowed them to more effectively learn the physical exam; 88% of students agreed with the statement “visualizing anatomy by ultrasound gave me more confidence in my physical exam skills.” Ninety-four percent of students felt that the US component should continue in the physical exam course. In addition, 91% of students agreed or strongly agreed that US should be given additional time throughout the four-year medical school curriculum. Table 3 shows average student responses.

### Advanced ultrasound selective

Twelve students participated in the three-hour US “selective.” All students completed a pre- and post-assessment survey. All students were able to correctly list the standard four views that make up the FAST examination following the session. Additionally, when shown an image of the right upper quadrant (Figure), no students were able to correctly identify the three structures prior to the session, while 11/12 students correctly identified all three structures in the post-assessment survey. All students increased their confidence in their ability to perform both a FAST exam as well as a focused cardiac exam following the session. Following the sessions, all 12 students agreed or strongly agreed that US skills are important to learn during medical school.

## DISCUSSION

As POCUS has taken a more prominent and diverse role throughout medical and surgical specialties, there has been increasing interest in introducing it earlier in medical training. Several studies have shown bedside ultrasonography to be a feasible addition to medical school education with a handful of schools reporting successful integration of a vertical curriculum over four years.^4^,^11^

Currently the Liaison Committee on Medical Education (LCME) does not include POCUS as mandatory for medical student education; however, it is clear that various technologies and digital resources have changed the way that students learn. Just as e-learning, simulation, and the instructional methodology of the “flipped classroom” has made its way into medical school education, POCUS has great potential to add blended learning to optimize student learning and retention. Furthermore, early exposure to learning US skills will help prepare students for future clinical work.

There have been multiple reports demonstrating that students’ understanding of anatomy and physical exam skills improve with the incorporation of US. Students also improve specific physical exam skills such as measuring liver size and detecting cardiac murmurs with the addition of focused ultrasound.^21^–^24^ Dinh et al recently reported their findings that a first-year curriculum into a physical diagnosis course may improve overall physical examination skills.^25^

Our initial ultrasound pilot program integrated into the first- and second-year curriculum for the 2013–14 academic year was well received by students. For a small subset of 12 students who took an advanced selective during the second year, a brief three-hour session may improve both confidence in performing exams as well as knowledge of image acquisition and interpretation. Throughout the pilot program, students overwhelmingly desired additional US sessions.

Despite positive student feedback, many challenges remain in the introduction of POCUS education into the medical school curriculum. Others have described limitations of time, space, financial resources, as well as trained faculty.^4^,^17^ At our institution, we are fortunate to have expertise in POCUS from a variety of specialties and only through a multidisciplinary effort involving emergency medicine, radiology, internal medicine, anatomy and physiology, cardiology, physical medicine and rehabilitation, and rheumatology have our initial efforts been successful. In a review of other programs, our effort seems to be unique in the number of disciplines actively involved in the planning and teaching efforts. Faculty time is often scarce and it took considerable effort to find well-trained, enthusiastic instructors to keep our student-to-instructor ratio at the goal of 4:1. To expand efforts in the pre-clinical as well as clinical years, future training of instructors is necessary. Focused “train-the-trainer” sessions led by expert POCUS faculty for residents and fellows interested in teaching, which occurred prior to the medical student sessions, allowed us to expand the number of our instructors as well.

We faced similar limitations in financial resources as described at other institutions as well. Our medical school does not yet own any US machines. Thus, we relied largely on equipment from other departments and in-kind use of equipment through vendor sources to meet the needs for the student sessions. Significant time and effort was required to arrange enough US systems for each session. Lack of equipment available in between sessions limits the opportunity for students to pursue self-directed learning for further reinforcement. Furthermore, access to US machines is limited on medical and surgical floors in the hospitals. For students to retain and use skills learned early in training, US machines must be available to students in clinical rotations. Similarly, trained faculty in POCUS, while expanding, remains limited across our clinical sites. In order to fully grow as a program, we must continue to advance knowledge and skills across all of our four affiliated hospitals.

We also faced challenges defining the most appropriate fit for our US curriculum and continue to better define the best fit as our program matures. Time in the medical student curriculum is limited and there are many competing interests. US programs may be offered as electives rather than core components of the curriculum.^26^ While still working to define the best fit and areas for growth for the US curriculum, this pilot program was successful only through significant open and collaborative dialogue between the ultrasound core faculty and many members of the Harvard Medical School faculty.

This effort was successful only after considerable discussion on multiple levels within Harvard Medical School, from individual course directors, course planning committees, and the dean of medical education. Only through initiating discussion across many hospital and multiple levels of curriculum development, were we able to obtain initial success for this pilot program. While attending large curriculum planning meetings was helpful to create an initial presence in the medical school, it was equally important to meet with and identify individual course directors to find time and space in the curriculum for our sessions. As we develop, we continue to engage educators at multiple levels within the medical school curriculum as well as at the various hospitals affiliated with the medical school. All such efforts are done in parallel as we hope to expand on our initial success to involve more hospitals as well as a greater presence in the four-year curriculum.

## LIMITATIONS

Our results are limited by the subjective nature of the data. Our outcomes using rating scales from student questionnaires are inherently limited. Future work should focus on observed skills and knowledge in the context of ultrasound education. We realize the subjective nature of our results are limited and hope to expand on initial efforts to examine stronger outcomes of students’ skills and competency from the introduction of US into the medical school curriculum. Due to financial and time constraints both of faculty as well as limited time in the student curriculum, we were unable to develop more objective outcome measures in this pilot study. We hope to develop a more substantial and objective evaluation process, which is essential as curricula develop and expand. As curricula mature and are more fully integrated into undergraduate medical education, there remains the need for guidelines to help focus future work.

The costs associated with an ultrasound program, from faculty time, time in the curriculum, as well as costs of machines, are substantial. To convince administrators the costs are worthwhile, we do hope to participate in future work examining the skills, knowledge, and ultimately improved clinical care that may come from the introduction of an ultrasound curriculum.

Furthermore, while a single institution and results are limited to our school, students at Harvard have courses at four primary hospitals and our efforts did involve discussion with multiple pre-clinical and clinical sites. We did only introduce the US sessions to a single site as part of the second-year physical exam course further limiting our experiences in the second-year curriculum. We also relied on multiple levels of instructors from residents to attending-level providers from a variety of specialties. While we worked hard to standardize each lesson plan, further work is needed to ensure a high quality of consistent teaching across all sessions. Despite these limitations, we feel our efforts offer lessons to other programs at early stages of developing an ultrasound curriculum in medical school education.

## CONCLUSION

Our pilot efforts have shown that integration of bedside ultrasonography into the pre-clinical medical school curriculum is well received by students. We used didactics and small group hands-on teaching sessions led by a multidisciplinary team of instructors to introduce ultrasound sessions into the medical school curriculum. Medical students perceived the US curriculum as valuable in better understanding human anatomy and learning physical exam skills. Within our pilot study, students uniformly expressed the desire for an expanded ultrasound curriculum. Further work aims to collect more objective data to guide national guidelines as further ultrasound programs develop and mature in medical student education.

## Figures and Tables

**Figure:**
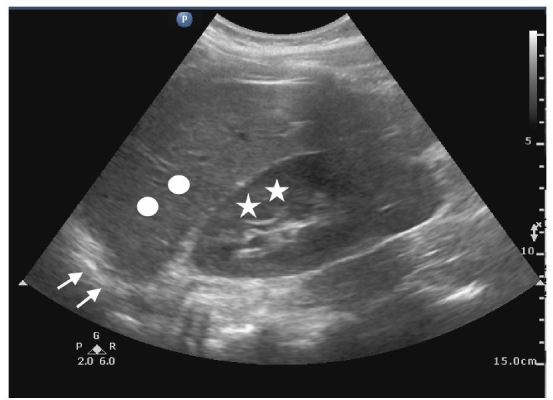
Assessment of students’ ability to identify basic structures in right upper quadrant. Liver marked with circle, kidney with stars, and diaphragm with arrows.

**Table 1:** Ultrasound curriculum for PGY (postgradutae year)-1 anatomy lab sessions.

Session	Objective
1. Neck	Identify carotid artery, jugular vein, and the thyroid; sono-anatomic difference between internal jugular vein and carotid artery
2. Cardiac	Identify basic cardiac views and orientation of heart chambers and valves
3. Abdomen	Identify relationship and orientation of liver, gallbladder, kidney, Morison’s pouch, diaphragm, spleen, aorta, vena cava
4. Musculoskeletal	While included joints and tendons of shoulder and elbow, due to time constraints the focus was placed on joints and tendons of the hand and digits, such as metacarpophalangeal joint, metacarpal bones, phalanx bones, flexor and extensor tendons.

**Table 2:** Ultrasound curriculum for PGY (postgraduate year)-2 physical exam course.

Session	Ultrasound skill objective	Physical exam skill objective
1. Introduction to ultrasound	Introduction to machine, basic terminology, transducer types, basic scanning techniques, orientation, and planes of view Sonographic appearance of fluid, soft tissue, air, bone, vessels, and distinguish arterial from venous vessels	Basic approach to distinguishing arteries from veins
2. Abdominal ultrasound	Demonstrate and visualize ultrasound appearance of liver, kidney, gallbladder, spleen, bladder, bowel, diaphragm, aorta, vena cava	Examine and percuss liver and spleen borders, assess for Murphy’s sign, palpate aorta
3. Neck and thyroid ultrasound	Evaluate normal and abnormal thyroid ultrasound, carotid artery, jugular vein, arterial and venous waveforms	Palpate borders of thyroid, assess jugular venous pressure
4. Musculoskeletal ultrasound	Demonstrate and visualize ultrasound appearance of muscle, tendon, bone, nerve Perform physical exam maneuvers while visualizing bones, tendons, nerves, joints (shoulder, hand, wrist, knee, and ankle)	Inspection, palpation and physical exam maneuvers of the shoulder, knee, and ankle

**Table 3:** Average post-session PGY (postgraduate year)-2 student responses on scale from 1–5 (reported with standard deviation) given after physical exam course incorporating ultrasound.

Assessment question	Response
I would like to see ultrasound integrated into my medical education	4.52 (0.62)
Ultrasound has the ability to enhance my medical training in the pre-clinical courses	4.45 (0.62)
Ultrasound has the ability to enhance my medical training in the clinical years	4.67 (0.6)
I would benefit from continued ultrasound exposure throughout all four years of medical school	4.39 (0.74)
It is important for me to learn basic ultrasound skills during medical school	4.61 (0.56)
The addition of ultrasound to the physical diagnosis curriculum helped me more effectively learn physical exam skills	4.36 (0.92)
Visualizing anatomy by ultrasound gave me more confidence in my physical exam skills	4.33 (1.0)
Ultrasound should continue to be a part of the physical diagnosis course in the future	4.55 (0.67)
I would like to see ultrasound given more time throughout all four years of medical school	4.36 (0.82)

1=Strongly disagree

2=Disagree

3=Neither agree or disagree

4=Agree

5=Strongly agree
